# Geometry design for a fully insertable glucose biosensor with multimodal optical readout

**DOI:** 10.1117/1.JBO.27.11.117001

**Published:** 2022-11-18

**Authors:** Jesse Fine, Gerard L. Coté, Michael J. McShane

**Affiliations:** a Texas A&M University, Department of Biomedical Engineering, College Station, Texas, United States; b Texas A&M University, Center for Remote Health Technologies and Systems, Texas A&M Engineering Experiment Station, College Station, Texas, United States; c Texas A&M University, Department of Materials Science and Engineering, College Station, Texas, United States

**Keywords:** biophotonics, spectroscopy, fluorescence, phosphorescence, lifetime, wearable sensors, implanted devices

## Abstract

**Significance:**

Insertable optical continuous glucose monitors (CGMs) with wearable readers are a strong option for monitoring individuals with diabetes. However, a fully insertable CGM requires a small form factor while still delivering sufficient signal to be read through tissue by an external device. Previous work has suggested that a multimodal repeating unit (barcode) approach may meet these requirements, but the biosensor geometry must be optimized to meet performance criteria.

**Aim:**

This work details *in silico* trials conducted to evaluate the geometry of a fully insertable multimodal optical biosensor with respect to both optical output and species diffusion *in vivo*.

**Approach:**

Monte Carlo modeling is used to evaluate the luminescent output of three presupposed biosensor designs based on size constraints for an injectable and logical placement of the bar code compartments. Specifically, the sensitivity of the luminescent output to displacement of the biosensor in the X and Y directions, overall size of the selected design, and size of an individual repeating unit are analyzed. Further, an experimentally validated multiphysics model is used to evaluate the diffusion and reaction of glucose and oxygen within the biosensor to estimate the occurrence of chemical crosstalk between the assay components.

**Results:**

A stacked cylinder multimodal biosensor 4.4 mm in length with repeating units 0.36 mm in length was found to yield a greater luminescent output than the current “barcode” biosensor design. In addition, it was found that a biosensor with enzymatic elements does not significantly deplete glucose locally and thus does not impact the diffusion profile of glucose in adjacent compartments containing nonenzymatic assays.

**Conclusions:**

Computational modeling was used to design the geometry of a multimodal, insertable, and optical CGM to ensure that the optical output and chemical diffusion profile are sufficient for this device to function *in vivo*.

## 1 Introduction

Since the development of the first biosensor in modern history, a biological sample glucometer by Clark and Lyons in 1962,^1^ biosensors have expanded to be a ubiquitous component of acute and chronic disease management. In 2020, biosensors had grown to a global market size of $22,400,000,000 USD and were the largest number of commercial medical devices.^1^^,^^2^ With biosensors providing useful diagnostic and prognostic information related to physiological parameters, many diseases, such as cardiovascular disease and diabetes, can be treated and managed.^3^^,^^4^ A growing research space is that of continuous and skin-integrated biosensors. Often referred to as implantable, insertable, or injectable, these devices rest on or within a patient and enable precision medicine by providing real-time measurement of physiological biomarkers with minimal inconvenience to the patient.^5^^,^^6^ Due to its prevalence, morbidity, and mortality, diabetes has been the focus of much research for continuous biosensors and personalized/precision medicine.^7^^,^^8^ Continuous glucose monitors (CGMs) have been proven to improve diabetes management and reduce negative outcomes affiliated with disease progression, and they can provide care to the over 122 million American adults that are estimated to be diabetic or prediabetic.^9^^–^^11^ Moreover, although CGMs were first approved by the Food and Drug Administration (FDA) in 1999, recent work is being done to integrate CGMs holistically into diabetes management to supplement essential interventional components, such as diet monitoring.^12^^,^^13^ Thus, as CGMs become more integral to diabetes management best practices, it is key to continue research and development in new CGM devices.

Although becoming more integral to diabetes management, current commercial CGMs—with the three market leaders being Dexcom (Dexcom G6), Medtronic (Medtronic Guardian/MiniMed 670G), and Abbott (Abbott Freestyle Libre Pro)—are relatively expensive, create risk for infection, and have a short lifetime.^14^^,^^15^ The only fully implantable glucose biosensor, Senseonic’s Eversense, requires a physician to perform implantation and explantation and is cost prohibitive. As described previously, several researchers are developing an inexpensive, multimodal, and fully insertable glucose biosensor, which is unique in its small form factor (6.5  mm×0.7  mm×0.9  mm, enabling injection through a hypodermic needle).^16^^,^^17^ In addition, this biosensor has repeating unit functionality, wherein two separate assays independently provide glucose predictions, and optical signal transduction, which enables a combination of the biosensor’s recognition and transduction elements. Specifically, this biosensor uses a phosphorescence lifetime decay within an enzymatic schema (assay 1) and Förster resonance energy transfer within a competitive binding schema (assay 2). Previous work demonstrated the use of the Monte Carlo (MC) method to establish that the anticipated optical signal strength of this biosensor is great enough to be detected by a photodiode as a significant concern with optical and indwelling biosensors is the inability of the signal to propagate through tissue.^18^

However, there remains a need to further consider the geometry of this biosensor to maximize performance, namely modifications to the form factor to increase optical output and ensure satisfactory chemical functionality within the subcutaneous space is of great importance. Computational modeling is a valuable tool for evaluating biosensor geometry as *in silico* methods can provide accurate results more efficiently with respect to time and cost when properly verified and validated. Specifically, MC methods have frequently been used to estimate photon propagation through turbid media.^19^^–^^21^

There is also a need to validate and create a model that can be used to estimate reaction kinetics and molecular interactions between the biosensor and the *in vivo* environment as a function of the biosensor geometry. Specifically, whether the two assays interact chemically need to be investigated to explore whether the luminescent output of one assay is affected by the diffusion gradients or local conditions created by the other assay. This model does not currently exist; thus, we present the use of COMSOL Multiphysics to build such a model and validate its use experimentally. Multiphysics platforms, such as COMSOL^®^, are effective tools for evaluating complex systems, such as biosensors, due to their ability to simulate complex conditions.^22^ For example, multiphysics platforms can use computational fluid dynamics methods to evaluate the deformation of a biosensor from fluid flow and understand how that deformation affects the diffusion profiles of species into the biosensor.^23^^,^^24^ Here, we present the use of MC modeling to evaluate luminescent output of a biosensor as it depends on geometry, as well as develop and validate a multiphysics model to evaluate chemical functionality.

## 2 Materials and Methods

### 2.1 MC Model

#### 2.1.1 Model geometry and properties

**Fig. 1:**
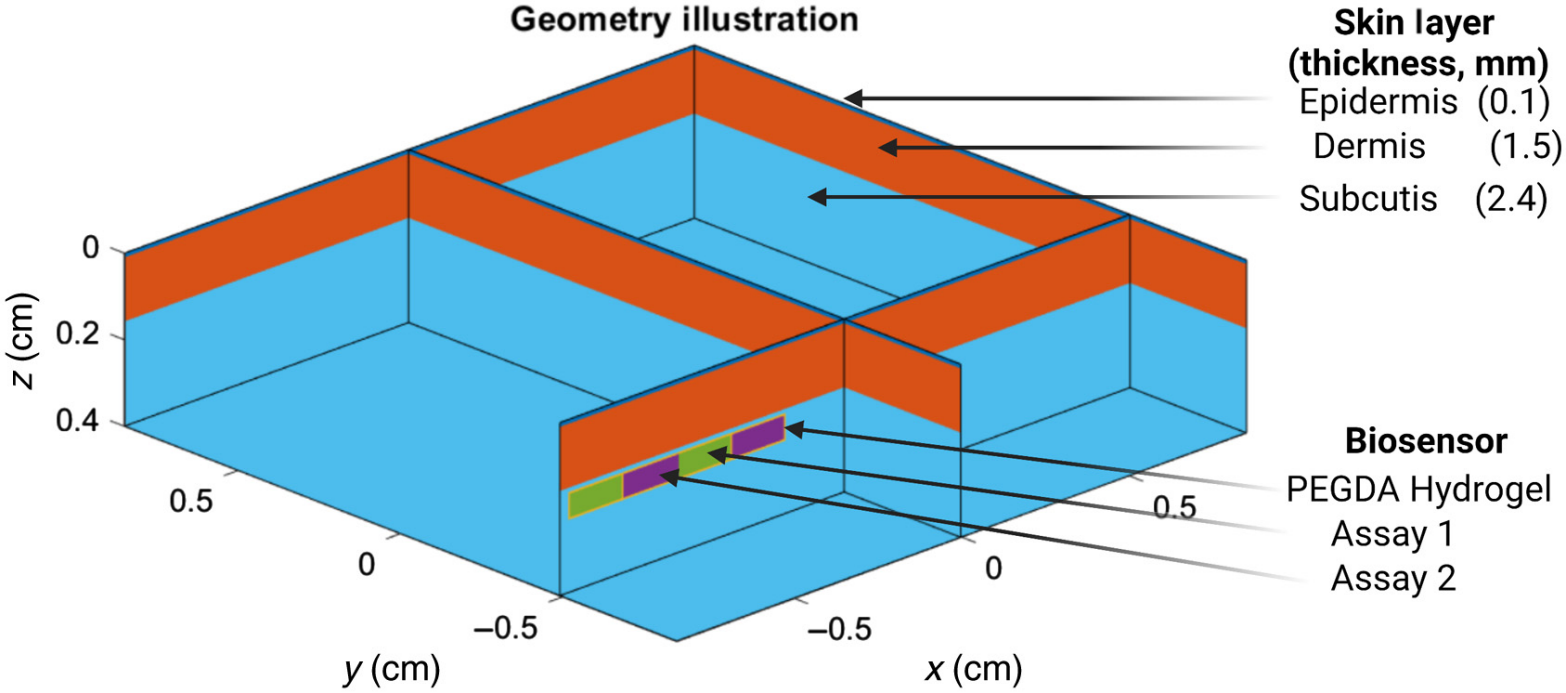
MC model geometry.

The MC modeling of photon propagation is a scientifically appropriate option for evaluating the biosensor size, design, and subsequent impact on luminescent output. Thus, an MC model created via MCmatlab, previously described elsewhere, was used within this work.^25^ In short, a three-layer skin model (epidermis, dermis, and hypodermis) of the dorsal wrist 17  mm×17  mm×4  mm (length × width × height) was created, as shown in Fig. 1. Optical properties for tissue were derived from Jacques et al.,^26^ and thickness values were derived from Chen et al.,^27^ Sandby-Moller et al.,^28^ and Van Mulder et al.^29^ Optical properties used for this model are found in Table 1. The refractive index for all components is set to 1 for refractive index matching, which is a needed step to simulate geometries that are heterogenous in the Z direction within MCmatlab.^21^ Within this tissue model is a biosensor of variable location and size, as described in Sec. 2.1.2. The simulated light source is located at the origin (0,0,0) and is light emitting diode (LED)-like with a top-hat distribution in the near field and a Lambertian distribution in the far field with a side length of 1.75 mm and a wavelength of 680 nm. These parameters were chosen to match commercial offerings from ThorLabs single-color visible LEDs because these are assumed to be high-probability options for designing the associated wearable in future work. Luminescent emission was simulated at 800 nm, and the power yield of the fluorophores was set to 1 to enable a comparison of results between simulations while minimizing computational time. Each voxel was a 0.05-mm cube. Each simulation was repeated three times with $1e^{7}\text{ photons}$, which was shown to be sufficient through parametric analysis from previous work.^17^ To better analyze the biosensor response, the saved output of each simulation is the emission light that reached the top surface of the geometry as opposed to a photodiode or detector. All simulations were completed in MATLAB 2019b on a Lenovo Legion Y720b (Windows 10) and were parallelized to an NVIDIA GeForce GTX 1060.

**Table 1:** The MC model optical properties.

Feature	$\mu_{a}$ (680/800) ${\mathrm{cm}}^{-1}$	$\mu_{s}$ (680/800) ${\mathrm{cm}}^{-1}$	n	g	Quantum yield
Epidermis	0.78/0.46	294.1/250.0	1	0.9	0
Dermis	0.48/0.30	195.3/140.8	1	0.9	0
Poly(ethylene glycol) diacrylate hydrogel	${1e}^{-6}/{1e}^{-6}$	${5e}^{-4}/{5e}^{-4}$	1	0.75	0
Assay 1	${5/1e}^{-6}$	${1e}^{-6}/{1e}^{-6}$	1	0.75	1
Assay 2	${5/1e}^{-6}$	${1e}^{-6}/{1e}^{-6}$	1	0.75	1

#### 2.1.2 MC simulations

**Fig. 2:**
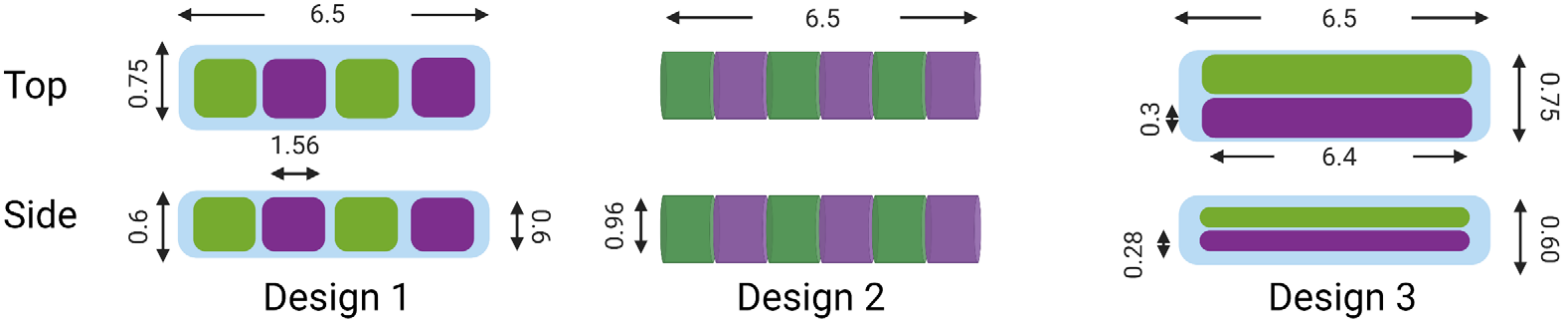
Presupposed biosensor designs (mm). Green and purple domains represent alternating sensing assays. Created with Ref. 30.

A simulated biosensor was inserted within the tissue model described in Sec 2.1.1 to evaluate luminescent performance of both assays under different conditions. As shown in Fig. 2, three different designs were considered. Each sensor was required to have the following design constraints: (1) be 6.5 mm in length, (2) have repeating units for the two sensing assays, (3) enable 17-gauge hypodermic needle insertion, and (4) have visible areas with both sensing chemistries across its four sides. These designs were tested to quantify overall luminescent output by simulating photon interactions in the case in which the center of the biosensor rests at 0  mm×0  mm×2  mm and all emission light reaching the top surface of the skin is assumed to be captured. However, in practice, patient motion will potentially result in offsets being between the center illumination source axis and the insertable biosensor axis. Thus, sensitivity analyses of these designs to offsets are required.

To conduct an analysis of the biosensor luminescent output sensitivity to displacement between the illumination source and the biosensor, simulations were conducted wherein each iteration of the biosensor was centered at 5, 3, 0, −3, and −5  mm in the X and Y dimensions as well as 1.25, 2, and 2.75 in the Z dimension. This work determined which biosensor has the least variation in luminescent output as a function of X, Y, and Z dimension offsets and overall which biosensor design yields the greatest luminescent output.

The next step is to assess the length of the longest dimension and the thickness of the individual repeating unit for the biosensor design. The length of the optimized biosensor can be found by analyzing the full width half maximum (FWHM) of excitation light that originates from the LED at the target implant depth of 2 mm. Then, the ideal thickness of a single sensing compartment was found by completing iterations of simulations wherein the 6.5-mm biosensor is divided into 1, 3, 5, 7, 9, 11, 13, and 15 compartments of each assay and located 5 mm in the positive and negative X and Y axes. It is hypothesized that the luminescent output of these simulations will eventually converge once the number of each sensing assays increases to a given amount. The assay compartment thickness wherein luminescent output converges is the optimal suggested size of the sensing compartment because then it can be said that a typical excitation beam will be exciting both assays relatively equally. This prevents one assay from failing due to insufficient excitation. From these MC simulations, the final output is a biosensor that will be determined based on its strong luminescent output with respect to the limitation of being able to be inserted via a hypodermic needle.

### 2.2 COMSOL Multiphysics Model

#### 2.2.1 Validation of species diffusion, reaction, and phosphorescence decay

A two-dimensional multiphysics model (validation model) was constructed in COMSOL Multiphysics 5.2 (COMSOL, Inc., Sweden) to (1) represent the diffusion of glucose and oxygen into a phosphorescence lifetime decay-based glucose biosensor and (2) represent the reaction of glucose and oxygen. Subsequently, this model also demonstrated the quenching of a phosphorescent dye, such as Pd-meso-tetra (sulfophenyl) tetrabenzoporphyrin (HULK, Frontier Scientific), by calculating the phosphorescence lifetime as a function of the Stern–Volmer relationship ($k_{\mathrm{sv}}=0.023$ and $\tau_{0}=201\;\mu s$).^31^ The validation model was constructed to represent the testing of phosphorescence lifetime decay enzymatic biosensors within a “flow-through” system as described elsewhere and was performed with a single sample biosensor.^32^^,^^33^ Briefly, the biosensor itself consists of alginate microparticles contained within an alginate hydrogel. Within the microparticles are HULK, glucose oxidase (GOx, 146.2 mmol, *Aspergillus niger*, BBI Solutions, Cardiff, Wales, United Kingdom), and Catalase (0.61 mol, bovine liver, Calzyme Laboratories, Inc., San Luis Obispo, California, United States). Using layer-by-layer assembly, the diffusive properties of this gel can be controlled such that, when *in vivo*, the diffusive rate of glucose and oxygen into the sensor is within a known range.^34^ As oxygen enters the biosensor, the oxygen dynamically quenches the biosensor; however, the presence of glucose causes the consumption of oxygen via enzymatically driven glucose oxidation, as described in the following equations:

$$E+G↔X_{1}\to E^{\prime }+\text{gluconate},$$ (1)

$$E^{\prime }+O_{2}↔X_{2}\to E+H_{2}O_{2},$$ (2)

where E is oxidized GOx, G is glucose, $X_{1}$ is enzyme-substrate complex 1, $E^{\prime }$ is reduced GOx, and $X_{2}$ is enzyme-substrate complex 2.^35^ The reaction rate for the reversible formation of enzyme-substrate complex 1 is $k_{1}$ and $k_{-1}$, the reaction rate for the formation of gluconate and reduced GOx is $k_{2}$, the reaction rate for the reversible formation of enzyme-substrate complex 2 is $k_{3}$ and $k_{-3}$, and the reaction rate for the formation of hydrogen peroxide is $k_{4}$.

Thus, the phosphorescence lifetime of the biosensor may be indirectly correlated to glucose. The flow-through system is a bench top evaluation system wherein sensors are placed in Delrin channels with glass windows, and a glucose/phosphate-buffered saline solution is pumped through at rates and concentrations controlled by LabVIEW (National Instruments, Austin, Texas, United States). A version of this system used for lactate sensing is described and illustrated in Ref. 32. Directly above the sensor is a “reader head,” which excites with a pulsed LED and collects phosphorescence emission from the biosensor at a high sampling frequency, enabling the determination of phosphorescence lifetime by fitting the exponential decay.

**Fig. 3:**
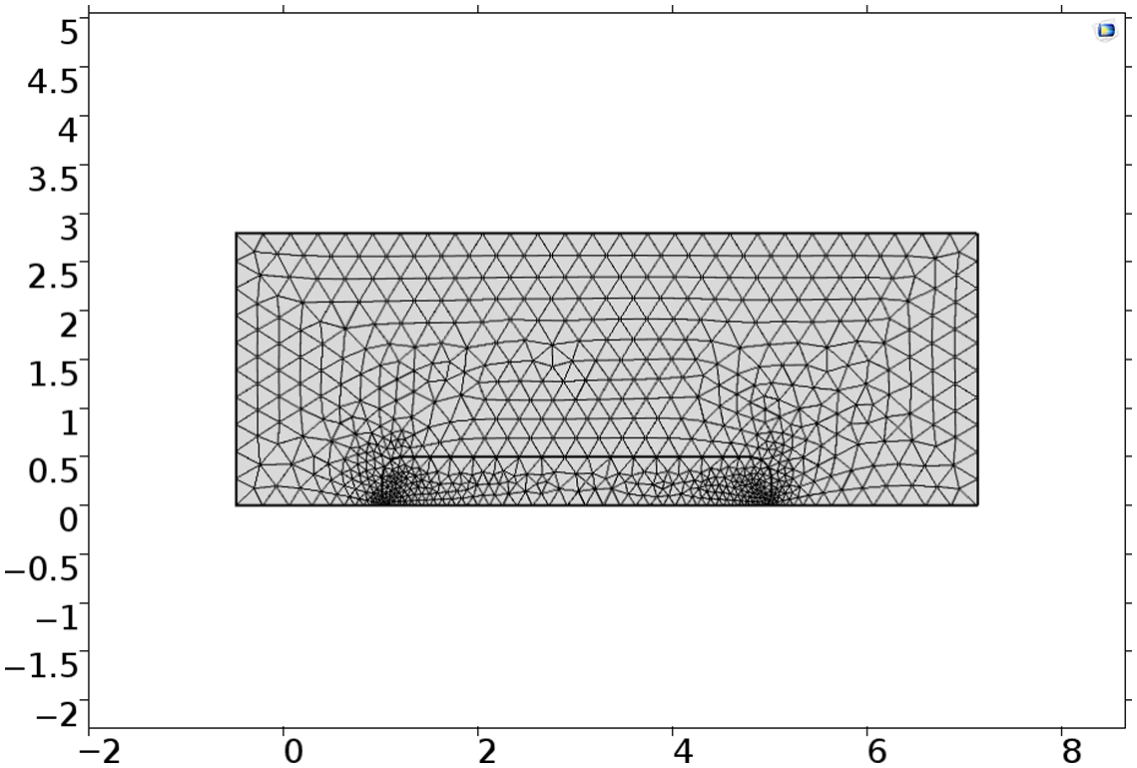
Meshed COMSOL cross-section model of flow-through system (cm).

The COMSOL representation of this testing system is limited to the flow cell chamber wherein the biosensor rests, as shown in Fig. 3. The geometry of the vertical cross section is a 7.6  mm×2.8  mm (length × height) rectangle with a 4  mm×0.5  mm (length × height). The biosensor is centered on the bottom surface with a geometrical fillet to replicate the cylindrical representation of the biosensor. The glucose solution enters from the left boundary with a velocity of 0.009  m/s and exits through the right boundary via the laminar flow COMSOL physics module. The chemical transport and reaction properties of this biosensor are viewable in Table 2, and the reaction rates are described as^35^

$$\frac{\partial G}{\partial t}=D_{G}\frac{\partial^{2}G}{\partial z^{2}}-k_{1}GE+k_{-1}X_{1},$$ (3)

$$\frac{\partial O_{2}}{\partial t}=D_{O}\frac{\partial^{2}O_{2}}{\partial z^{2}}-k_{3}O_{2}E^{\prime }+k_{-3}X_{2},$$ (4)

$$\frac{\partial E}{\partial t}=-k_{1}GE+k_{-1}X_{1}+k_{4}X_{2},$$ (5)

$$\frac{\partial E^{\prime }}{\partial t}=k_{2}X_{1}-k_{3}O_{2}E^{\prime }+k_{-3}X_{2},$$ (6)

$$\frac{\partial X_{1}}{\partial t}=k_{1}GE-(k_{-1}+k_{2})X_{1},$$ (7)

$$\frac{\partial X_{2}}{\partial t}=k_{3}O_{2}E^{\prime }-(k_{-3}+k_{4})X_{2}.$$ (8)

**Table 2:** Biosensor diffusion and reaction properties.

Parameter	Value	Unit	Description	References
$k_{1}$	100	$m^{3}/(s*\mathrm{mol})$	Reaction rate	35
$k_{-1}$	0.003	$s^{-1}$	Reaction rate	35
$k_{2}$	300	$s^{-1}$	Reaction rate	35
$k_{3}$	1000	$m^{3}/(s*\mathrm{mol})$	Reaction rate	35
$k_{-3}$	150	$s^{-1}$	Reaction rate	35
$k_{4}$	50	$s^{-1}$	Reaction rate	35
$D_{G\_S}$	$2.64e^{-10}$	$m^{2}/s$	Diffusion of glucose in subcutaneous space	36
$D_{O2\_S}$	$1.5e^{-9}$	$m^{2}/s$	Diffusion of oxygen in subcutaneous space	37
${\mathrm{IC}}_{G}$	2.78	$\mathrm{mol}/m^{3}$	Initial concentration of glucose	38
${\mathrm{IC}}_{O2}$	0.009	$\mathrm{mol}/m^{3}$	Initial concentration of oxygen	39
$R_{G}$	−0.027	$\mathrm{mol}/(m^{3}*s)$	Reaction of glucose consumption by cells	40 and 41
$R_{O2}$	$-1.04e^{-5}$	$\mathrm{mol}/(m^{3}*s)$	Reaction of oxygen consumption by cells	42
Et	0.1463	$\mathrm{mol}/m^{3}$	Enzyme concentration	n/a
Pw(t)	Figure 5	$\mathrm{mol}/m^{3}$	Concentration of glucose from capillaries at time t	38 and 43
$C_{O2}$	0.009	$\mathrm{mol}/m^{3}$	Constant concentration of oxygen from capillaries	39
$D_{G\_\mathrm{Assay}\;1}$	$4.0e^{-10}$	$m^{2}/s$	Diffusion of glucose in hydrogel	35
$D_{O2\_\mathrm{Assay}\;1}$	$2.45e^{-9}$	$m^{2}/s$	Diffusion of oxygen in hydrogel	35

To maximize computational efficiency and obtain a large average element quality, the biosensor was modeled with a triangular mesh with element sizes between 0.004 and 0.037 mm. The remainder of the domain—the “flow-cell”—is a triangular mesh with element sizes between 0.001 and 0.282 mm. The final average element quality or skewness is defined as a scalar measure of equiangular skew, where a value of 0 represents poor element size regularity that will prevent model convergence and a value of 1 is ideal. The skewness value was found to be sufficiently high at 0.9531.

To validate the ability of this model to represent (1) diffusion/reaction of species and (2) biosensor quenching and phosphorescence lifetime, simulation and real experiments modeling exposure to different glucose concentrations were performed. Specifically, five different concentrations ranging from 0 to $11.10\;{\mathrm{mol}/m}^{3}$ (0, 2.78, 5.55, 8.34, and $11.1\;{\mathrm{mol}/m}^{3}$) were used as inputs as they cover the physiological range of glucose concentration for individuals with diabetes.^44^ These concentrations with four biosensors were completed and replicated *in silico*. To represent biosensor quenching and phosphorescence lifetime, the lifetime output of the model was compared with the experimental lifetime in three biosensors at each glucose concentration. To compare the diffusion/reaction of the species, the time to steady-state (TSS) lifetime of the experiment and computational counterpart, defined as the time between 5% greater than the initial lifetime and 5% of the next steady-state lifetime, were also compared.

#### 2.2.2 Simulated biosensor performance when inserted

**Fig. 4:**
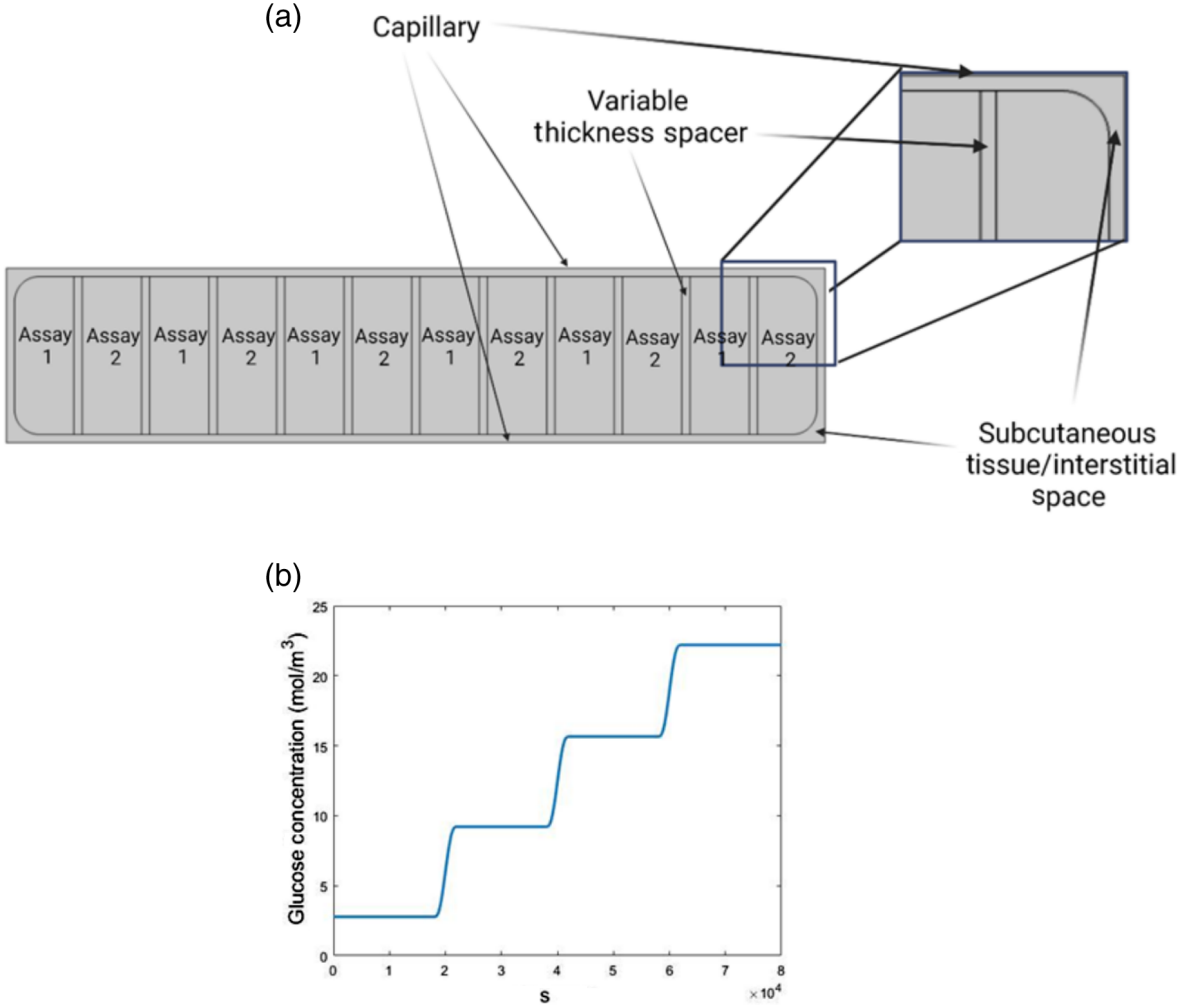
(a) COMSOL multiphysics model of interstitial space and biosensor and (b) capillary glucose concentration as a function of time “pw(t).”

A second COMSOL model, representing the interstitial space ∼2  mm below the surface of the volar forearm, was developed with information sourced from the literature. This model geometry is shown in Fig. 4. The model is 1 mm in height, and the length is determined by the size of the biosensor being simulated, which was variable in this case and ranges from 4.53 to 15.42 mm. This was done to simulate biosensors with variable “spacers” from 0.01 to 0.1 cm while maintaining the domain thickness of 0.036 cm to evaluate the impact of the glucose diffusion profile caused by the enzymatic sensing domains on the nonenzymatic domains. The sensing domains have triangular mesh elements ranging from 0.0001 to 0.05 mm in size, and the remainder of the geometry has triangular mesh elements ranging in size from 0.006 to 0.184 mm. Overall, this yields an average element quality from 0.94 to 0.98. In this simulation, glucose and oxygen are sourced from the top and bottom boundaries—representing capillaries—that are 0.05 mm from the biosensor in each direction, as determined by the capillary density in the subcutis. Glucose has a variable concentration within the physiological range, and oxygen is supplied at $0.009\;{\mathrm{mol}/m}^{3}$. In addition to the glucose and oxygen consumption that occurs by the biosensor, the background tissue is consuming these species in the form of a 0th order reaction with rates −0.027 and $-1.{04e}^{-5}\;\mathrm{mol}/(m^{3}*s)$, as shown in Refs. 40–42. To evaluate the impact of the glucose diffusion profile caused by the enzymatic biosensor on the nonenzymatic domains, the concentration of glucose was evaluated within the nonenzymatic domains for all biosensors with different spacer sizes.

## 3 Results and Discussion

### 3.1 MC-Based Biosensor Geometry Optimization

**Fig. 5:**
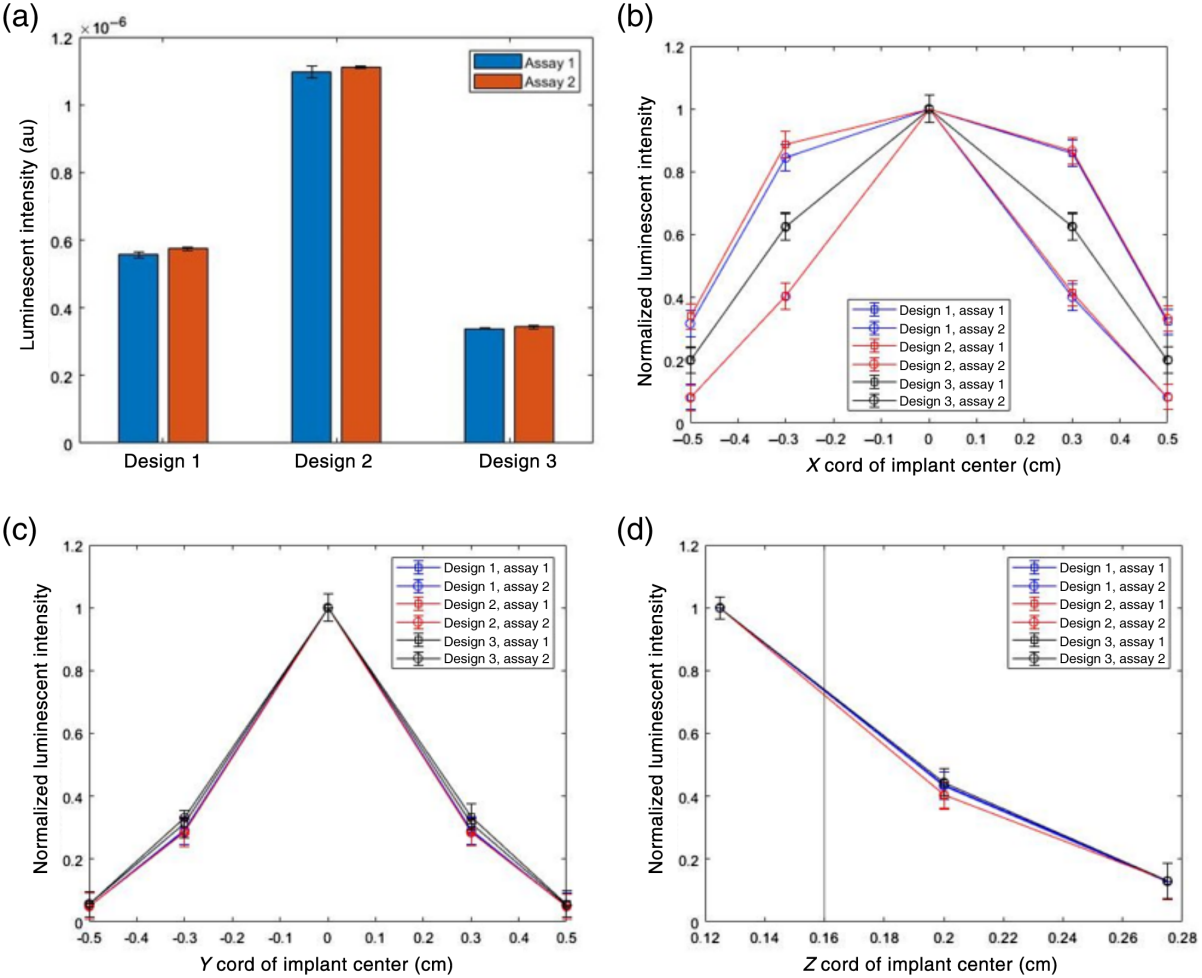
(a) Luminescent intensity of three presupposed designs, (b) sensitivity of relative luminescent intensity of three designs in the X axis, (c) sensitivity of relative luminescent intensity of three designs in the Y axis, and (d) sensitivity of relative luminescent intensity of three designs in the Z axis. Error bars are 1 standard deviation. Vertical line at X=0.16  cm in panel (d) indicates the position (depth) of the dermal/subcutaneous junction. Error bars are 1 standard deviation.

Key parameters in choosing the best performing biosensor geometry are overall luminescent intensity and sensitivity of the intensity as a function of relative changes in position between the excitation source and the biosensor in all three dimensions. Figure 5(a) shows the luminescent output of both assays for the three presupposed designs in arbitrary units. On average, design 2 yields the greatest luminescent output, being 1.97 times the luminescent output of design 1 and 3.22 times the luminescent output of design 3. This is possible due to the cylindrical shape of design 2, which enables it to have a greater volume and still meet the size requirement of insertion with a hypodermic needle. Figures 5(b)–5(d) indicate the relative luminescent intensity of each biosensor design when translated 10 mm in the X direction (−5 to 5 mm), 10 mm in the Y direction (−5 to 5 mm), and 1.6 mm in the Z direction (1.2 to 2.8 mm). In the Y and Z direction, each biosensor design performs similarly. All designs experience an ∼70% reduction in luminescent intensity as they are shifted 3 mm in the Y direction and a 95% reduction in luminescent intensity as they are shifted 5 mm in the Y direction. The required rotational symmetry of the biosensors is one reason for the symmetric drop in luminescent intensity being observed in the positive and negative Y coordinates. It was anticipated that designs 1 and 3 would be more robust than design 2 in this sensitivity analysis as both have surfaces that are perpendicular to the excitation light; however, this was not found to be the case. In the Z direction, the intensity decreases ∼70% as implantation depth increases from 1.2 to 2 mm and 90% as implantation depth increases from 1.2 to 2.8 mm. As expected, all designs perform similarly. In the X direction, designs 1 and 2 have an assay that retains 90% of luminescent intensity between −3 and 3 mm, whereas design 3 has a relative luminescent intensity of 75% at −3 and 3 mm. In addition, at a maximum displacement of −5 and 5 mm, designs 1 and 2 have an assay that retains 40% of their maximum luminescent output. This is because designs 1 and 2 have repeating units along the long axis of the biosensor, whereas design 3 has longer and fewer repeating units that extend over the length of the biosensor. This allows designs 1 and 2 to maintain strong luminescent output of at least one assay over 6 mm of displacement. This is a favorable outcome compared with design 3 because it is not likely that the biosensor and wearable will be consistently colocated when movement of the wearable and biosensor occur. Overall, it was found that designs 1 and 2 have comparably similar performances in the sensitivity analysis; however, the overall luminescence in design 2 is the greatest, and subsequently design 2 is the strongest option. However, it can still be further optimized; specifically, the overall length of the biosensor and the length of individual repeating units can be optimized to maximize luminescent output.

**Fig. 6:**
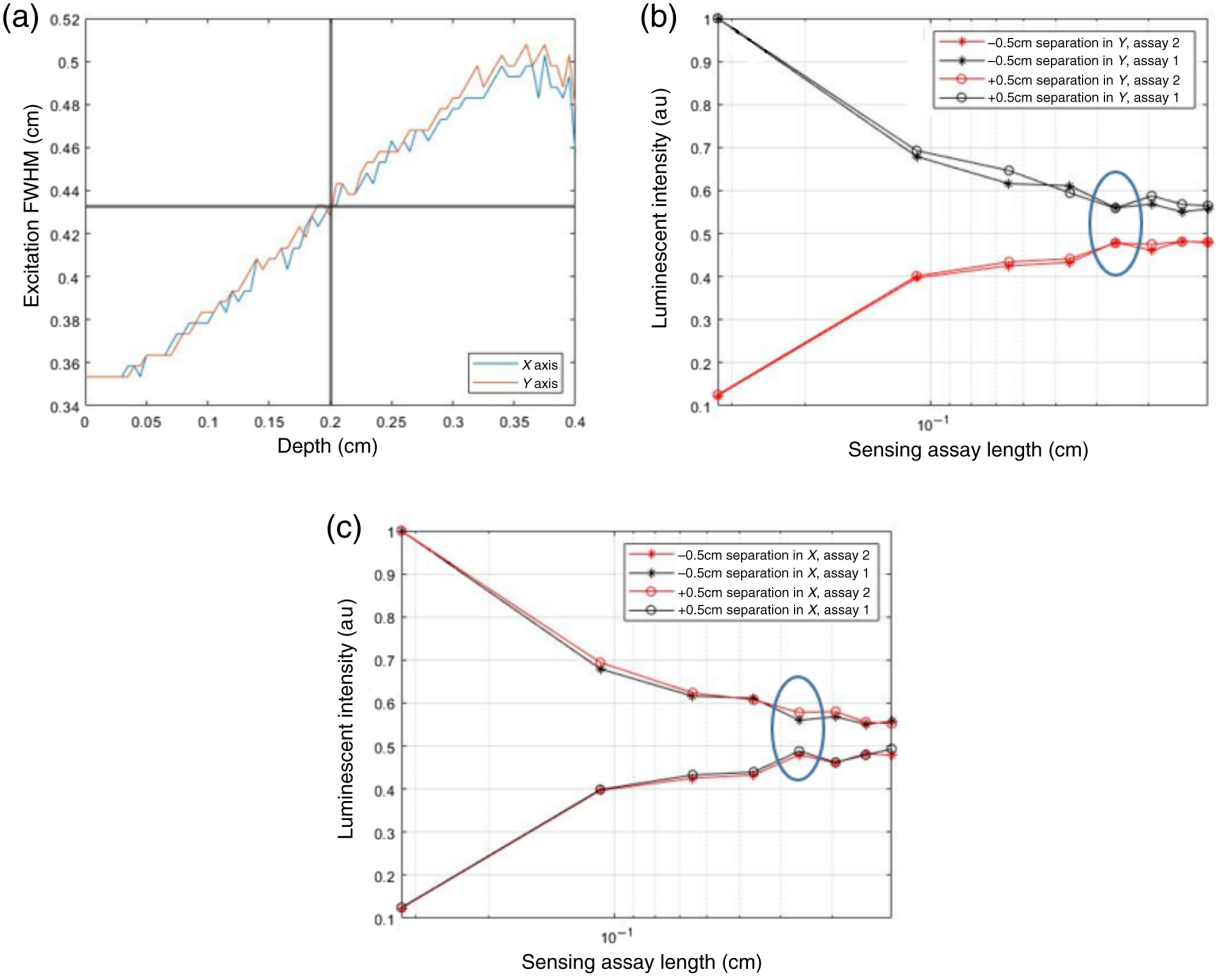
(a) FWHM of an LED in tissue, (b) luminescent intensity versus sensing assay length for displacement in the Y axis, (c) luminescent intensity versus sensing assay length for displacement in the X axis. The blue circles on panels (b) and (c) indicate the optimal size.

The ideal overall length of the biosensor is the FWHM of the excitation beam at the target insertion depth of 2 mm. This ensures that the biosensor is long enough to make full use of the LEDs excitation despite divergence, but not too long such that some of the biosensor remains unexcited. To determine this ideal length, MC simulations were carried out with the same geometry and properties as the previous component of this work, which analyzed the profile of excitation light within the geometry. At a depth of 2 mm, it was found that the FWHM in the X and Y directions was 4.3 mm, as shown in Fig. 6(a). Thus, for this simulated light source, the ideal biosensor length should be 4.3 mm. To determine the ideal length of one repeating unit, and consequently the total number of repeating units, 64 MC simulations with a biosensor placed with −5 and 5 mm offsets in the X and Y directions for each of the two assays were completed. The biosensor geometry was changed such that the number of repeating units in the biosensor was varied from 3.25 to 0.21 mm. The results of these simulations, as shown in Figs. 6(b) and 6(c), illustrate that, if the biosensor is only subdivided into one of each sensing compartment and the biosensor is not directly under the excitation light, one of the two assays will not strongly luminesce, depending on the positioning of the biosensor. As the unit thickness of a repeating unit is decreased and more total repeating units are included, the luminescence outputs of both assays converge. It was then found that using 0.36-mm-thick repeating units allows enough domains of each assay to exist such that luminescence is largely consistent across sensing chemistries. This was determined because there is a <5% change in luminescent intensity as the repeating unit thickness decreases. This leads to a final compartment thickness of 0.36 mm and 12 total sensing chemistry domains within a 4.3-mm biosensor.

### 3.2 COMSOL Multiphysics Biosensor Analysis

**Fig. 7:**
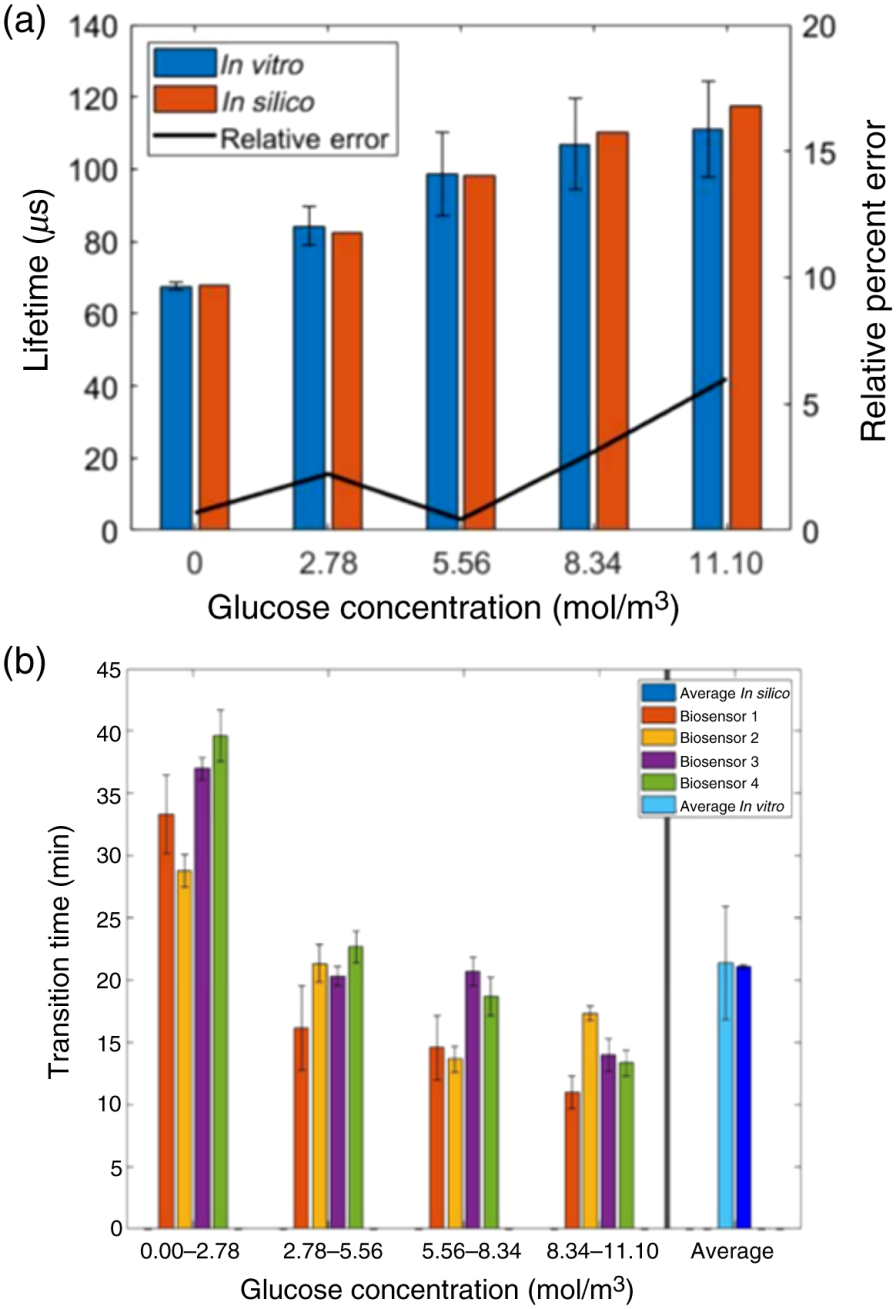
Validation of (a) species reaction and (b) species diffusion. Error bars are 1 standard deviation. Created with Ref. 30.

To first predict biosensor performance *in vivo* with a computational model, a valid model must be established. Here, the model was validated by matching the simulated phosphorescence lifetime of a biosensor within the phosphorescence lifetime in an experimental flow cell, confirming the capability to simulate phosphorescence lifetime as well as diffusion and reaction of oxygen and glucose. Figure 7(a) illustrates the comparison of lifetimes measured in computational and experimental work from $0\;\mathrm{mol}/m^{3}$ of glucose to $11.1\;\mathrm{mol}/m^{3}$ of glucose. Overall, the average percent error of the simulated lifetime and experimentally determined lifetime is 2.5%, ranging from 0.70% when the glucose concentration was 0  mg/dL to 6.28% when glucose concentration was 200  mg/dL. This is represented by the black bar in Fig. 7(a). With the strong agreement between the experimental phosphorescence lifetimes and their *in silico* counterparts, the representation of glucose oxidation in this computational model is validated. However, an important consideration is the speed at which glucose and oxygen diffuse within the biosensor. Thus, further work needs to be done to validate the ability of this model to represent the diffusive properties of an *in vivo* insertable biosensor.

Figure 7(b) illustrates the comparison of the transition time that it took for the experimental setup to change lifetimes as glucose concentration changed and the time that it took for the simulations to perform the same change. *In vitro*, four biosensors had a TSS of 21.41 min as the flowing glucose concentration was changed in four even intervals from 0 to $11.1\;\mathrm{mol}/m^{3}$. *In silico*, the average TSS was found to be 21.08 min. This agreement is very strong; however, the *in vitro* data exhibit a negative trend in TSS as glucose concentration increases, which was not found in the *in silico* data. It is hypothesized that this negative trend is a result of a shell effect wherein the glucose and oxygen will have access to more phosphors once the outer layer of particles is quenched. However, given that this mechanism is a result of phosphorescence quenching properties as opposed to diffusion properties, the agreement in average TSS is taken to mean the model is able to recreate diffusion of glucose and oxygen within the biosensor. Thus, with a validated computational model, the diffusive gradient of the biosensor can be explored to determine if the enzymatic sensing domain negatively impacts the glucose concentration that reaches the nonenzymatic sensing domains.

**Fig. 8:**
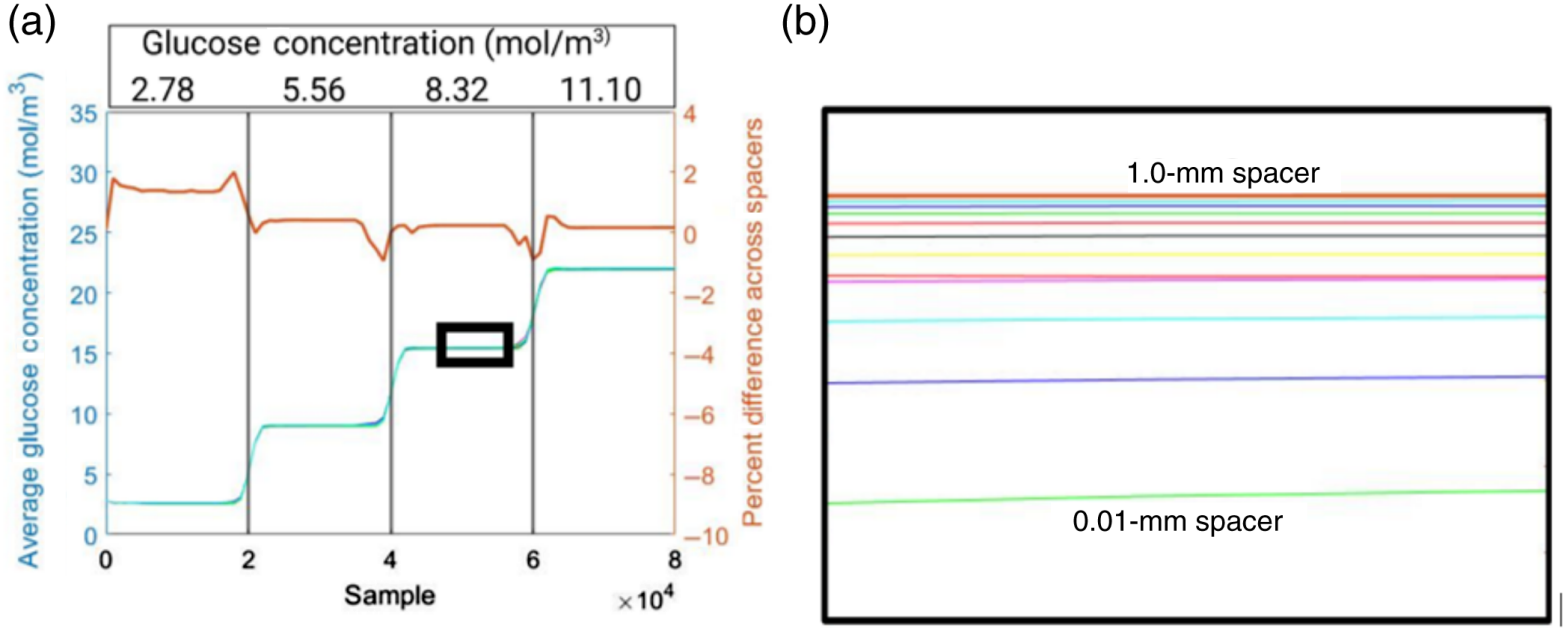
(a) Effect of variable thickness spacer (spacer) size on glucose concentration. Vertical bars indicate the center of glucose concentration transition. (b) Magnified view of the inset from panel (a). Created with Ref. 30.

Figure 8(a) depicts the average glucose concentration observed in each of the six nonenzymatic sensing domains for all biosensor versions with different spacer sizes. These data are enlarged in Fig. 8(b). On the right axis of Fig. 8(a) is data detailing the percent difference in glucose concentration across the 1- and 0.01-mm spacer. The percent difference ranges from −1% to 2% and thus is not likely to significantly affect the functionality of the nonenzymatic sensing domains in any case.

## 4 Conclusion

This work is an example of using multiple advanced modeling methods to design medical devices. Specifically, an MC model of photon propagation and a COMSOL multiphysics model of diffusion–reaction were developed and used in tandem to design a geometry for a fully insertable and multimodal optical biosensor for glucose monitoring. This is the first case, to the authors’ knowledge, of specifically utilizing multiple computation models to design an insertable chemooptical device. The MC simulations were used effectively to evaluate and optimize different geometric designs, yielding a barcode with an ideal total length of 4.3 mm with 0.36-mm-thick repeating units. Multiphysics diffusion–reaction models were also able to reliably predict sensor behavior and were used to further conclude that there is no anticipated chemical crosstalk between reaction regions of the barcoded biosensor. Due to the complexity of the computational modeling within this work, experimental validation and verification should be pursued both to validate the findings presented herein and to better understand how factors such as optical property variability affects these results. Whereas this work features the exploration of a geometry for a fully insertable biosensor, the design of the associated wearable should also be explored. Specifically, the layout of LEDs and photodiodes will affect the amount of luminescent output that is received by the wearable device.

## Data Availability

The code utilized and data generated for the completion of this work can be supplied by the authors upon reasonable request.
